# Evolution in a plant matrix: adaptive reshaping of kefir grains microbiota and function during long-term soymilk culture

**DOI:** 10.3389/fmicb.2025.1614639

**Published:** 2025-06-04

**Authors:** Zhina Chen, Qingqing Li, Fanqi Li, Linlin Yin, La Wang, Tao Ye, Yi Wang, Shengju Fu, Weiming Wang, Xiaochen Huang

**Affiliations:** ^1^School of Biological Engineering, Huainan Normal University, Huainan, China; ^2^School of Food and Pharmaceutical Engineering, Zhaoqing University, Zhaoqing, China

**Keywords:** kefir grains, microbial succession, soymilk, subculture, *Lacticaseibacillus paracasei*

## Abstract

To explore the adaptability of kefir grains in long-term subculture in soymilk, this study tracked the succession and functional changes of its microbial community over 4 months. High-throughput sequencing results showed that the microbial community structure was drastically reshaped, mainly manifested in the relative abundance of *Lacticaseibacillus kefiranofaciens* decreasing from 95.00 to 15.70%, while *Lacticaseibacillus paracasei* increased from 0.32 to 76.94%, becoming the dominant bacteria. Metagenomic analysis indicated that *L. paracasei* possesses key enzymes for metabolizing raffinose, stachyose and sucrose, which is the basis for its efficient utilization of soymilk oligosaccharides and its competitive advantage. The decrease in the abundance of *L. kefiranofaciens* was associated with a decrease in the synthesis of extracellular polysaccharides (EPS), which in turn caused a reduction in the diameter of kefir grains, an increase in surface viscosity and a partial collapse of the gel matrix structure. The pH and free amino acid content of fermented soymilk did not fluctuate much during the passage process, but the sensory acceptance, antioxidant capacity and angiotensin converting enzyme (ACE) inhibitory activity all showed a downward trend. This work reveals the adaptive evolution mechanism of kefir grains in a plant matrix environment and provides a theoretical basis for the optimization of soymilk fermentation based on limited strains.

## 1 Introduction

Kefir grains are a traditional milk starter culture originating from Caucasus, Mongolia and East Europe (Gökırmaklı and Güzel-Seydim, 2022). These gelatinous and viscous structures range in size from 0.3 to 3.5 cm, resemble small cauliflower florets, and exhibit colors ranging from white to yellow (Gut et al., 2019). Kefir grains consist of a natural matrix of exopolysaccharides (EPS), primarily kefiran and proteins within which lactic acid bacteria (LAB), yeasts, and acetic acid bacteria (AAB) co-exist in a symbiotic relationship (Garofalo et al., 2020). LAB commonly found in kefir grains include *Lactobacillus kefiranofaciens*, *Lentilactobacillus kefiri* (previously known as *Lactobacillus kefiri*), *Lactococcus lactis*, *Leuconostoc mesenteroides*, *Lactobacillus acidophilus*, *Levilactobacillus brevis* (previously known as *Lactobacillus brevis*), and *Lentilactobacillus parakefiri* (previously known as *Lactobacillus parakefiri*) among others (Alraddadi et al., 2023; Purutoğlu et al., 2020). On the other hand, yeasts such as *Saccharomyces cerevisiae*, *Kluyveromyces marxianus, Candida kefyr* and *Kazachstania unispora* (Azizi et al., 2021; Zanirati et al., 2015) are the predominant yeast species present in kefir grains. While the microbial communities in kefir grains can vary significantly based on regional and cultivation conditions (Barãoa et al., 2019; Gul et al., 2015), *Lactobacillus kefiranofaciens* is consistently reported as a predominant and stable species, maintaining high abundance and content during long-term propagation (Alraddadi et al., 2023; Wang et al., 2018; Zeng et al., 2022). However, the microbial succession observed when kefir grains are propagated in non-bovine dairy substrates demonstrates notable differences. For example, Wang et al. (2020) reported that the relative abundance of *L. kefiranofaciens* in kefir grains from the CN source decreased to 26.55% after a 2-months serial culture in goat milk but subsequently increased to became the most abundant species after 3 months of cultivation.

In recent years, the growing popularity of plant-based diets has led to the exploration of soymilk as a novel substrate for kefir fermentation (Baú et al., 2015; Egea et al., 2023; Liu and Lin, 2000; Luo et al., 2023), serving as a substitute for cow milk due to its advantages such as high protein content and low cholesterol. However, compared to cow milk, soymilk exhibits significant differences in its nutritional composition and physicochemical properties. The primary oligosaccharides in soymilk are sucrose, stachyose and raffinose, with almost no lactose (Baú et al., 2015), whereas lactose is the predominant carbohydrate in cow milk. This disparity may influence succession of microbial communities within kefir grains during long-term propagation in soymilk, which in turn, could affect the fermentation characteristics and overall quality of the resulting soymilk-based kefir.

Therefore, elucidating the succession of microbial communities in kefir grains during soymilk propagation and its influence on fermentation characteristics is crucial for the development of high-quality soymilk-based kefir products. This study aims to employ high-throughput sequencing technology to analyze the composition and structural dynamics of kefir microbial communities during propagation in soymilk. At the same time, combined with physical and chemical indicators and sensory evaluation, the specific effects of microbial succession on fermentation characteristics were explored, aiming to lay a theoretical foundation for the industrial production of soymilk-based kefir.

## 2 Materials and methods

### 2.1 Soymilk preparation

Soybeans (100 g) were screened, rinsed thoroughly with distilled water, and then soaked in distilled water at ambient temperature water for 8 h to facilitate hydration. The hydrated beans were then ground with distilled water at ratio of 1:7 (w/v). The resulting slurry was distributed into 250 mL Erlenmeyer flasks at a volume of 100 mL per flask and sterilized at 121°C for 20 min (Ketnawa and Ogawa, 2021). After cooling down to room temperature, the sterilized slurry was stored at 4°C pending subsequent experiments.

### 2.2 Passage and sampling of kefir grains in soymilk

Kefir grains were inoculated into 100 mL of sterile soymilk at an inoculation rate of 5% (v/v) and cultivated at 25°C under static conditions for 48 h. Following activation, the kefir grains were aseptically collected using a sterile stainless steel filter mesh and repeatedly washed with sterile deionized water to remove any residual soymilk from the grain surface. The washed kefir grains were subsequently transferred to fresh sterile soymilk medium at an inoculation rate of 5% (v/v) and incubated statically at 25°C, with the medium replaced every 48 h over a continuous subculture period of 4 months. Approximately 1 g of kefir grains samples were collected at 0 months (m0), 1 month (m1), 2 months (m2), and 4 months (m4) of subculture and stored at −80°C for metagenomic analysis.

### 2.3 Metagenomic technology analysis

Total microbial DNA was extracted from samples using the cetyltrimethylammonium bromide (CTAB) method with minor modification (Porebski et al., 1997). Approximately 0.5 g of kefir grains was frozen in liquid nitrogen and ground thoroughly to a fine powder using a pre-chilled sterile mortar and pestle. The powdered sample was transferred into a 2.0 mL microcentrifuge tube, and 800 μL of preheated CTAB extraction buffer was added. The mixture was incubated at 65°C for 30 min with occasional gentle mixing.

The lysate was sequentially extracted with equal volumes of phenol (pH 8.0): chloroform: isoamyl alcohol (25: 24: 1, v/v/v) and chloroform: isoamyl alcohol (24: 1, v/v), each followed by centrifugation at 12,000 rpm for 10 min. The aqueous phase was transferred to a new tube, mixed with an equal volume of cold isopropanol, and incubated at −20°C for 1 h to precipitate DNA.

Following centrifugation at 12,000 rpm for 10 min, the DNA pellet was washed twice with 75% ethanol, air-dried, and dissolved in sterile ddH₂O. To remove RNA, 1 μL of RNase A (10 mg/mL) was added and incubated at 37°C for 15 min.

The DNA was detected by 1% agarose gel electrophoresis (90 V, 30 min) stained with GelRed (Biotium, USA) and visualized under UV light. The DNA concentration and purity were determined with a NanoDrop 2000 spectrophotometer (Thermo Scientific, Wilmington, USA) at 260 and 280 nm and it was quantified using a Qubit® 2.0 Fluorometer (Thermo Scientific). The extracted microbial metagenomic DNA was randomly fragmented into short segments, and insert fragment libraries of appropriate lengths were constructed. Paired-end (PE) sequencing was performed on these sequences. Shotgun metagenomic sequencing was conducted by Shenzhen Microeco Technology Group Co., Ltd.

Raw sequencing data underwent quality control (via Trimmomatic) and host DNA removal (via Bowtie2) using the KneadData software, with FastQC employed to validate the rationality and efficacy of quality control. Taxonomic annotation of reads was performed using the Greedy mode of Kraken2. Subsequently, valid sequencing data were subjected to *de novo* assembly and redundancy removal to generate non-redundant contigs. Gene and protein prediction was then conducted based on the non-redundant contigs. Protein sequences were clustered to obtain a non-redundant protein sequence set, with the abundance of each protein sequence corresponding to its associated gene sequence. Transcripts Per Kilobase of exon model per Million mapped reads (TPM) was applied to quantify abundance. Functional and taxonomic annotation of each non-redundant protein sequence was achieved through alignment against the KEGG, NCBI, and NR databases. Finally, the correspondence between microbial community taxonomy and functional profiles was summarized based on the annotation results. Bioinformatics analysis and visualization were performed using the Microeco BioCloud Platform.^1^

### 2.4 Morphological transformations of kefir grains during sequential subculturing

#### 2.4.1 Macroscopic morphology

Kefir grains (m0, m1, m2, and m4) were aseptically separated from fermented soymilk using sterile stainless steel filter mesh. Grains were washed with sterile distilled water and transferred into autoclaved luminum weighing dishes in which sterilized paper towels were placed to remove excess water. Images of the kefir grains were captured with a Huawei smartphone (Huawei Technologies Co., Ltd., Shenzhen, China). From each group, ten kefir grains were randomly selected for diameter measurement using a vernier caliper (Shanghai Measuring and Cutting Tools Co., Ltd., Shanghai, China), and the average diameter was subsequently calculated.

#### 2.4.2 Microscopic morphology

The microscopic morphology of kefir grains was examined using scanning electron microscopy (SEM) following a modified protocol described by Gao et al. (2012). Briefly, kefir grains were rinsed repeatedly with sterile deionized water and transferred to 2.5 mL microcentrifuge tubes. The samples were fixed by immersion in 2.5% glutaraldehyde at 4°C for 4 h. Following fixation, samples were washed three times with 0.1 M phosphate buffer (pH 7.4). Gradient dehydration was then performed by sequential immersion in a graded series of ethanol solutions (40, 50, 60, 70, 80, and 90% ethanol in water, v/v) for 15 min each, followed by two 15-min washes with absolute ethanol. Residual ethanol was replaced with isoamyl acetate through two 15-min incubations. Any remaining isoamyl acetate on the grain surfaces was removed by rinsing with 0.1 M phosphate buffer (pH 7.4). The treated samples were flash- frozen at −80°C and lyophilized in a vacuum freeze-dryer until completely dry. The dried grains were sputter-coated with gold and imaged using a Schottky field emission SEM (SU8200, Hitachi High-Tech, Japan) to characterize their surface microstructure.

### 2.5 Fermentation dynamics of kefir grains during sequential subculturing

#### 2.5.1 pH of fermented soymilk

Kefir grains (1 g) from each of the four sampling time points were inoculated into sterile soymilk medium and cultured continuously for 24 h. The pH of the resulting fermented soymilk was then measured using a calibrated pH meter (PHS-3C, INESA Electronics (Group) Co., Ltd., Shanghai, China).

#### 2.5.2 Sensory evaluation of fermented soymilk

A trained sensory evaluation panel consisting of 30 participants (15 males and 15 females) was assembled. Panelists assessed sensory attributes, including color, aroma, taste, and texture, using a 100-point hedonic scale. Detailed evaluation criteria are presented in Table 1.

**Table 1:** Sensory evaluation standards for fermented soymilk.

Item	Evaluation criteria	Score range (points)
Color (20 points)	Uniform color, milky white with a glossy appearance	15 ~ 20
	Fairly uniform color, pale milky yellow	10 ~ 14
	Uneven color or discoloration	0 ~ 9
Aroma (15 points)	Pleasant aroma of lactic acid fermentation, no off odor	10 ~ 15
	Faint fermentation aroma or slight off odor	5 ~ 9
	No fermentation aroma, strong bean smell, off odor	0 ~ 4
Taste (35 points)	Smooth texture, balanced sour–sweet taste, lingering aftertaste	25 ~ 35
	Average taste, slightly unbalanced sour–sweet ratio, still acceptable	15 ~ 24
	Rough taste, too sour or too sweet, difficult to swallow	0 ~ 14
Texture (30 points)	Smooth surface, smooth, viscous texture, no whey separation	20 ~ 30
	Small holes on the surface, slightly thinner texture, minor whey separation	10 ~ 19
	Curd-like surface, thin texture, significant whey separation	0 ~ 9

### 2.6 Determination of amino nitrogen content

To quantify amino nitrogen content, a 10 mL aliquot of fermented soymilk was accurately transferred to a 100 mL volumetric flask and dilute to the volume with distilled water. A 25.0 mL aliquot of the diluted solution was then transferred to a 100 mL beaker and titrated with a standardized 0.05 M NaOH solution to a pH of 8.2, maintaining this pH for 1 min. Subsequently, 10 mL of formaldehyde solution (37% w/v) was added, the mixture was thoroughly agitated for 1 min, and the solution was titrated again with standardized 0.05 M NaOH solution to a pH of 9.2. The volume of NaOH consumed (V_1_) was recorded. A blank titration was performed concurrently, using 25 mL of distilled water in place of the sample, and the volume of NaOH consumed (V_2_) was recorded. The amino nitrogen content (*X*) in 100 mL of fermented soymilk sample was then calculated using Equation 1.


(1) $$X\;(\text{mg}/L)=(V_{1}-V_{2})\times M\times 14.007\times 1,000/\text{Volume of sample}$$

In this calculation, the molarity of NaOH solution is 0.05 M, and the atomic weight of nitrogen is 14.007 g/moL.

### 2.7 Determination of antioxidant and angiotensin-converting enzyme (ACE) activity

#### 2.7.1 2,2′-Azino-bis (3-ethylbenzothiazoline-6-sulphonic acid) assay (ABTS assay)

2,2′-Azino-bis (3-ethylbenzothiazoline-6-sulphonic acid) radical cation (ABTS^+^•) was generated by reacting a 7 mM ABTS stock solution with 2.45 mM potassium persulphate. This mixture was incubated in the dark at room temperature for 12 ~ 16 h before use (Chen et al., 2015).

The resulting ABTS•^+^ solution was then diluted with ethanol to achieve reached an absorbance of 0.70 ± 0.02 at 734 nm (Re et al., 1999). For the assay, 0.2 mL of sample (or distilled water for the control) was added to 2 mL of diluted ABTS•^+^ solution and incubated at 25°C for 30 min. The absorbance of each sample at 734 nm was subsequently measured using an ultraviolet–visible spectrophotometer (D-7PC, Runqee (Shanghai) Instruments Technology Co., Ltd., Shanghai, China). The ABTS•^+^ radical scavenging activity of the, expressed as the percentage inhibition (*I*%), was calculated using Equation 2.


(2) $$I\%=[(A_{\text{blank}}-A_{\text{sample}})/A_{\text{blank}}]\times 100$$

In this equation, A_sample_ and A_blank_ represent the absorbance of the reaction mixture with the test sample and the distilled water control, respectively.

#### 2.7.2 2,2-Diphenyl-1-picrylhydrazyl assay (DPPH assay)

A 0.5 mL aliquot of a 60 μM solution of DPPH in ethanol was mixed with 3.0 mL of sample (or distilled water for the control), homogenized by vortexing and incubated for 30 min in the dark at room temperature. Subsequently, the absorbance at 517 nm was recorded (Aspri et al., 2018). The DPPH radical scavenging activity of the test sample was calculated using Equation 2, employing the same method as described for the ABTS Assay.

#### 2.7.3 Ferric-reducing antioxidant power assay (FRAP assay)

The ferric-reducing antioxidant power (FRAP) assay was performed following the protocol described by Chen et al. (2015). Briefly, 0.1 mL of either standard or sample solutions was mixed with 2.45 mL of freshly prepared ferric-2,4,6-tripyridyl-s-triazine (ferric-TPTZ) reagent that prepared by mixing 300 mM acetate buffer (pH3.6), 10 mM TPTZ in 40 mM HCl and 20 mM FeCl_3_ at a volumetric ratio of 10:1:1. The resulting mixtures were incubated at 37°C for 10 min before measuring their absorbance at 593 nm. FRAP values were expressed as Trolox equivalents units of μg/mL.

#### 2.7.4 Angiotensin-converting enzyme (ACE) inhibitory activity

ACE inhibitory activity was determined according to the method described by Li et al. (2018). Briefly, 10 μL of ACE solution (0.25 U/mL, from rabbit lung) and 10 μL of sample or Tis-HCl buffer (used as the blank) were added to the wells of a 96-well microplate. The reaction was initiated by the addition of 150 μL of preheated (37°C, 5 min) substrate peptide FAPGG (1 mmol/L in 50 mmol/L Tris–HCl containing 0.3 mol/L NaCl, pH 7.5). The microplate was rapidly placed in the microplate reader, and the absorbance at 340 nm was recorded (A_1_). After a 30 min incubation, the absorbance was measured again at 340 nm (A_2_). The blank initial absorbance was recorded as A_01_, and the absorbance after the reaction was recorded as A_02_. The ACE inhibition rate was calculated based on the changes in absorbance, where A_inhibitor_ = A_1_-A_2_ and A_control_ = A_01_-A_02_. The percentage of ACE inhibition was then calculated using Equation 3.


(3) $$\text{ACE}\;\text{inhibition activity}(\%)=(1-A_{\text{inhibitor}}/A_{\text{control}})\times 100\%$$

### 2.8 Data analysis

All experiments were conducted in triplicate and Tukey’s test was applied for statistical analysis. Statistical significance was determined at *p* < 0.05.

## 3 Results and discussion

### 3.1 Microbial composition of kefir grains

**Figure 1:**
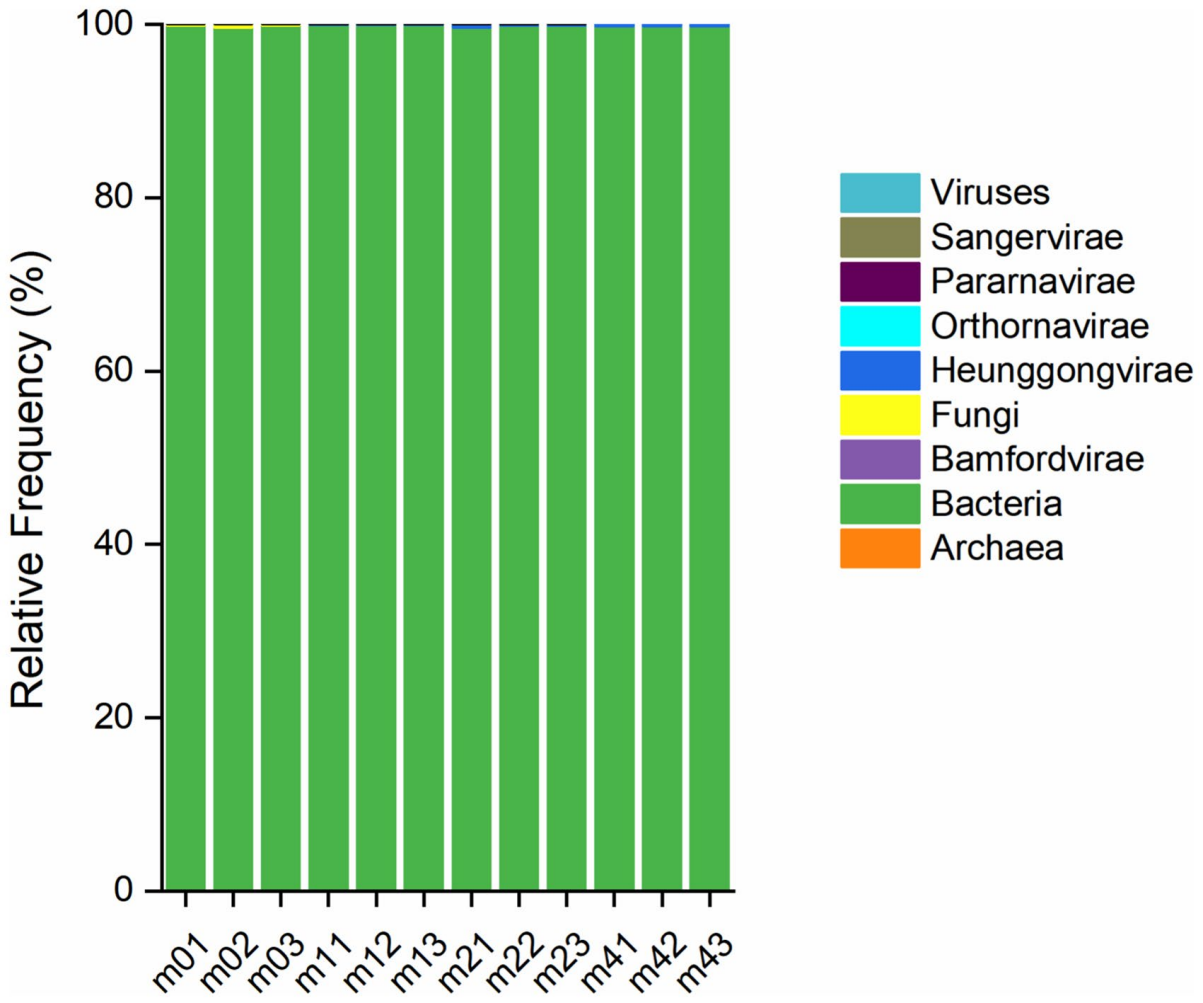
Relative taxonomic distribution of micoorganisms at the kingdom level in different kefir grains during subculturing (0 ~ 4 months) in soymilk. Samples m01 ~ m03, m11 ~ m13, m21 ~ m23, and m41 ~ m43 represent three replicates of kefir grains after 0, 1, 2, and 4 months of passage in soymilk, respectively.

Metagenomic sequencing and quality filtering (Q score ≥30) yielded 280,526,001 high-quality reads. Taxonomic profiling identified 9 biological domains. Bacteria dominated the community composition (99.83%), followed by Heunggongvirae (0.11%) and Fungi (0.06%). All other taxa were present at negligible levels, each accounting for less than 0.01% of the total community (as shown in Figure 1).

**Figure2:**
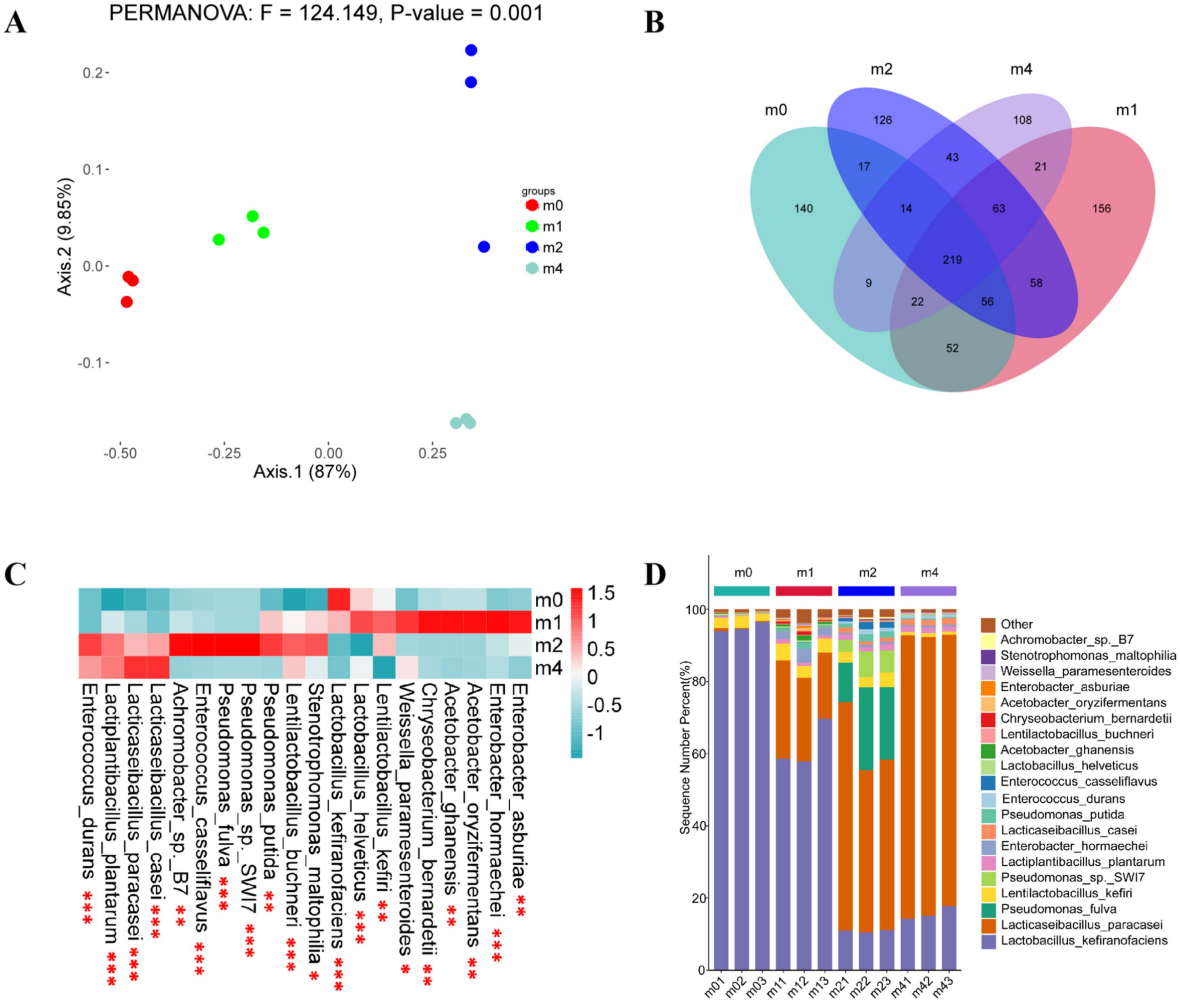
**(A)** Principal component analysis (PCA) ordination showing differences in the microbial flora of kefir grains during subculturing (0 ~ 4 months) in soymilk. Community dissimilarity was evaluated using the Unifrac distance metric. Principal components PC1 and PC2 explained 87.00 and 9.85% of community compositional variance, respectively; **(B)** Scalar-Venn representation of the same microbial species in kefir grains during subculturing (0 ~ 4 months) in soymilk; **(C)** Heatmap illustrating the distributing in abundances of the 20 most abundant strains within kefir grains during subculturing (0 ~ 4 months) in soymilk (*n* = 3). The log (strain abundance) is indicated by the color scale to the upper right and ranges from blue to dark red. Statistical significance levels: *p < 0.05, **p < 0.01, ***p < 0.001; **(D)** Proportions of dominant species (% of total community) in kefir grains during subculturing (0 ~ 4 months) in soymilk (*n* = 3). m0, m1, m2, and m4 represent kefir grains subjected to serial culture in soymilk for 0, 1, 2, and 4 months, respectively.

The principal component analysis (PCA) showed that PC1 and PC2 explained 87 and 9.85% of the variance, respectively, totaling 96.85% (Figure 2A). This indicates these two coordinates capture the vast majority of the variation in microbial community composition across samples. Therefore, these two principal coordinates were used as the basis for subsequent analyses (Long et al., 2022). The distinct clustering of the four groups clearly suggests significant differences in microbial community structure among the four kefir grains. A total of 529, 647, 596 and 499 species were identified in the m0, m1, m2, and m4 kefir grains, respectively (Figure 2B). Of these, 219 species were common to all four groups, representing 41.40% (m0), 33.85% (m1), 36.74% (m2) and 43.89% (m4) of the species richness in each respective community. Furthermore, the m0, m1, m2 and m4 communities contained 140, 159, 126 and 108 unique strains, respectively, which accounted for 26.47, 24.11, 21.14 and 21.64% of the species richness within each group.

A heatmap depicting the distribution and relative abundances of the 20 most abundant species across the four kefir grain communities is presented in Figure 2C. Although these top 20 species were common to all communities, their relative abundances differed significantly among the four kefir grains (indicated by asterisks). Figure 2D illustrates the changing proportion of dominant species relative to the total microbial community at each sampling time. As shown in Figure 2D, the dominant microbial community structure in kefir grains underwent substantial shifts with increasing subculture in soymilk. Prior to subculture (m0), *L. kefiranofaciens* was the overwhelmingly dominant species in the kefir grains, exhibiting a relative abundance of 95.00%, followed by *L. kefiri* (2.78%), *L. plantarum* (0.38%) and Lacticaseibacillus paracasei (0.32%). Consistent with our findings, previous studies have reported that *L. kefiranofaciens* typically accounts for over 80% of the total relative abundance in kefir grains, even across different sources (Alraddadi et al., 2023; Blasche et al., 2021; Wang et al., 2020; Wang et al., 2018; Zeng et al., 2022). Moreover, compared to other microbial species, *L. kefiranofaciens* exhibits strong resilience to changes in cultivation conditions, including substrate composition, drying processes, and non-sterile environments (Wang et al., 2018). For example, Blasche et al. (2021) demonstrated that *L. kefiranofaciens* maintains its dominance during continuous passage of kefir grains in cow milk by regulating kefir grain proliferation through metabolic activity and promoting adhesion via its synthesized polysaccharide matrix. However, contrasting with its stable dominance during animal milk-based fermentation, our study observed a decline in the relative abundance of *L. kefiranofaciens* to 62.04% after 1 month (m1) of serial passage in soymilk, accompanied by an increase in *L. paracasei* to 22.91%. After 2 months (m2), *L. kefiranofaciens* further decreased to 10.88%, while *L. paracasei* increased to 51.82%. By 4 months (m4), *L. kefiranofaciens* remained at 15.70%, while *L. paracasei* reached 76.94%, becoming the dominant species. In agreement with this trend, Mantegazza et al. (2023) isolated *Lactococcus lactis* subsp. lactis K03, *Leuconostoc pseudomesenteroides* K05, *Leuconostoc mesenteroides* K09 and *L. kefiri* K10, but did not detect *L. kefiranofaciens* after 14 consecutive passages using daily inoculation in fresh soy drink. These findings suggest that plant-based substrates may adversely affect the persistence of *L. kefiranofaciens* in kefir grain microbiota. This effect may be attributed to the limited availability of specific nutrients or growth factors present in dairy substrates, to which *L. kefiranofaciens* is better adapted, thereby reducing its competitive advantage in non-dairy environments.

**Figure 3:**
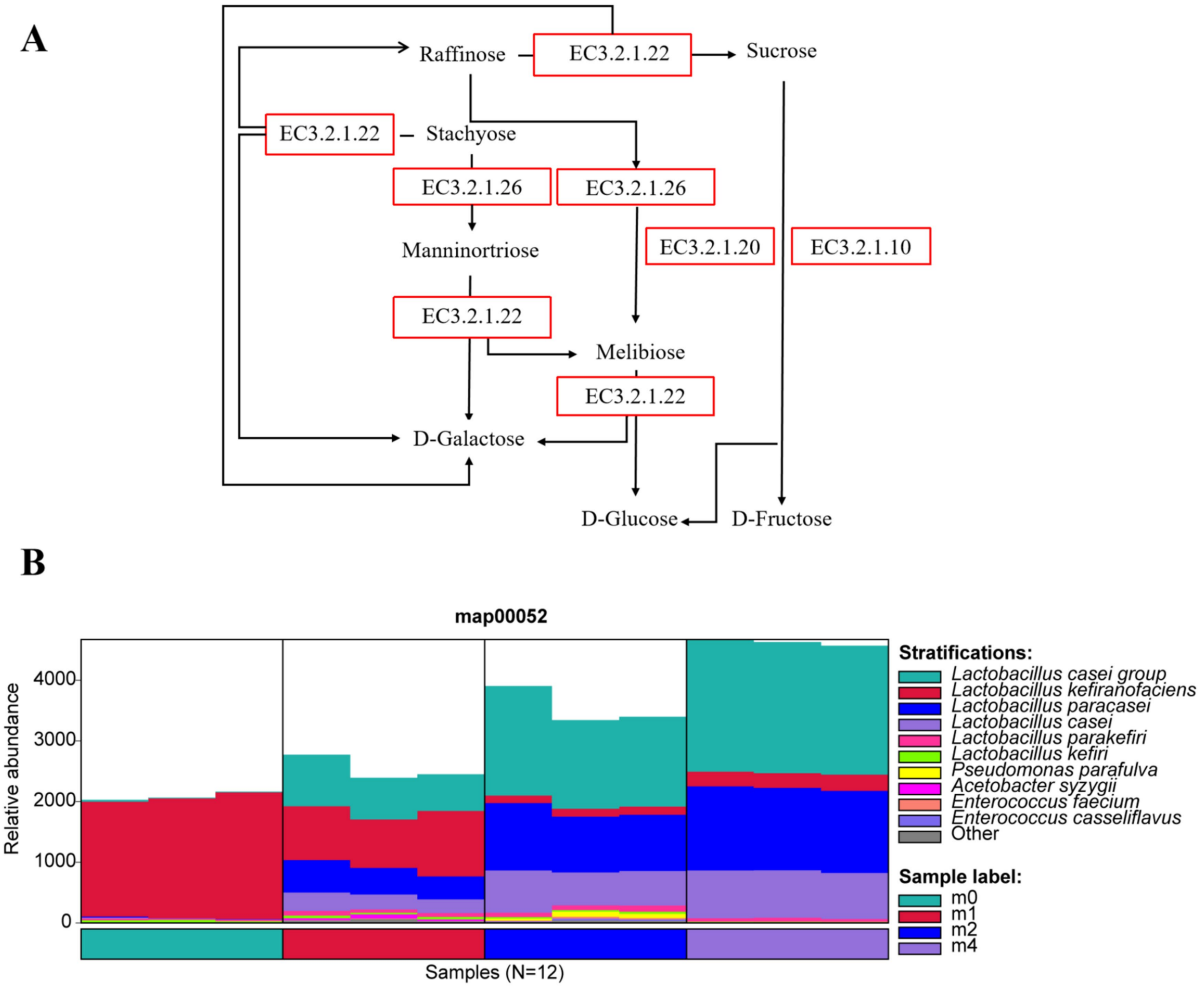
**(A)** Metabolic pathways of major soybean oligosaccharides (stachyose, raffinose, and sucrose) in soymilk, with enzymatic catalysis by *α*-galactosidase (EC 3.2.1.22), *β*-fructofuranosidase (EC 3.2.1.26), α-glucosidase (EC 3.2.1.20), and oligo-1,6-glucosidase (EC 3.2.1.10). **(B)** Succession of related microbial taxa in the MAP00052 lactose metabolism pathway of kefir grains during subculturing (0 ~ 4 months) in soymilk. m0, m1, m2, and m4 represent kefir grains subjected to serial culture in soymilk for 0, 1, 2, and 4 months, respectively.

During the serial subculture in soymilk, the relative abundances of *L. paracasei* and *L. casei* increased steadily, while *L. kefiranofaciens* consistently declined. We hypothesize that these shifts are driven by the distinct sugar profile of soymilk compared to bovine milk. Cow milk is predominantly lactose (98% of total sugars), whereas soymilk’s main sugar is sucrose (50%), followed by stachyose and raffinose (together 50%), with minor amounts of glucose and fructose (Mantegazza et al., 2023; Chen et al., 2025). Figure 3A shows the metabolic pathways for these soymilk sugars (stachyose, raffinose, and sucrose), primarily involving the lactose metabolism pathway (MAP00052) and key enzymes like *α*-galactosidase (EC 3.2.1.22), *β*-fructofuranosidase (EC 3.2.1.26), *α*-glucosidase (EC 3.2.1.20), and oligo-1,6-glucosidase (EC 3.2.1.10). The changes in the species associated with the MAP00052 pathway during soymilk passage are shown in Figure 3B. Initially (m0), *L. kefiranofaciens* dominated this pathway. However, with increasing passage time, its relative abundance fell while that of the *L. paracasei* group and *L. paracasei* itself rose, becoming the predominant species linked to this pathway by passage m4.

**Figure 4:**
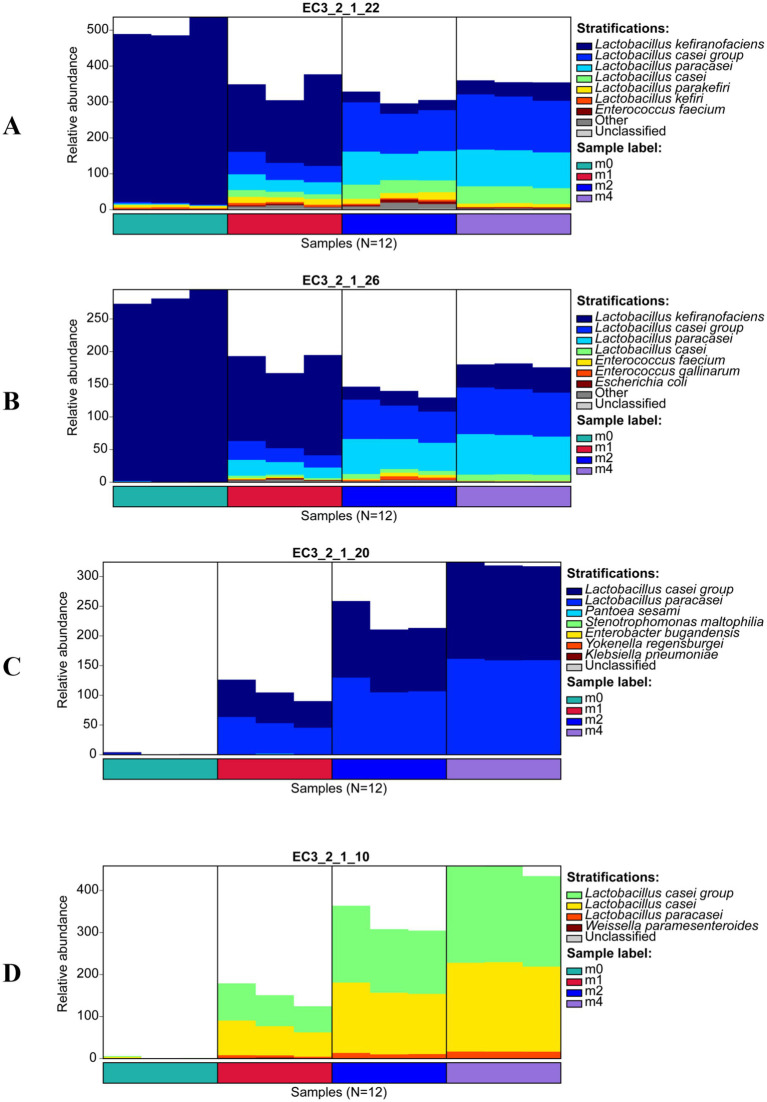
Microbial source succession of key carbohydrate-active enzymes of kefir grains during subculturing (0 ~ 4 months) in soymilk. **(A)** *α*-galactosidase (EC 3.2.1.22), **(B)** β-fructofuranosidase (EC 3.2.1.26), **(C)** *α*-glucosidase (EC 3.2.1.20), and **(D)** oligo-1,6-glucosidase (EC 3.2.1.10). m0, m1, m2, and m4 represent kefir grains subjected to serial culture in soymilk for 0, 1, 2, and 4 months, respectively.

Further analysis of the microbial sources of key enzymes (Figure 4) revealed that during serial passage, the primary microbial sources of *α*-galactosidase (EC 3.2.1.22) (Figure 4A) and β-fructofuranosidase (EC 3.2.1.26) (Figure 4B) shifted from *L. kefiranofaciens* to the *L. paracasei* group and *L. paracasei*. Notably, *α*-glucosidase (EC 3.2.1.20) (Figure 4C) and oligo-1,6-glucosidase (EC 3.2.1.10) (Figure 4D) were undetectable at m0, but after one month of passage (m1), these enzymes were primarily derived from *L. paracasei* group and *L. paracasei*, whose relative abundance continued to increase with further passage. These findings indicate that *L. paracasei* possesses key enzymes for the metabolism of raffinose, stachyose, and sucrose, demonstrating a strong capacity for utilizing these oligosaccharides. Consequently, *L. paracasei* gradually outcompeted *L. kefiranofaciens* during continuous passage, ultimately becoming the dominant species.

### 3.2 Morphological dynamics of kefir grains during subculture

#### 3.2.1 Macroscopic morphology

**Figure 5:**
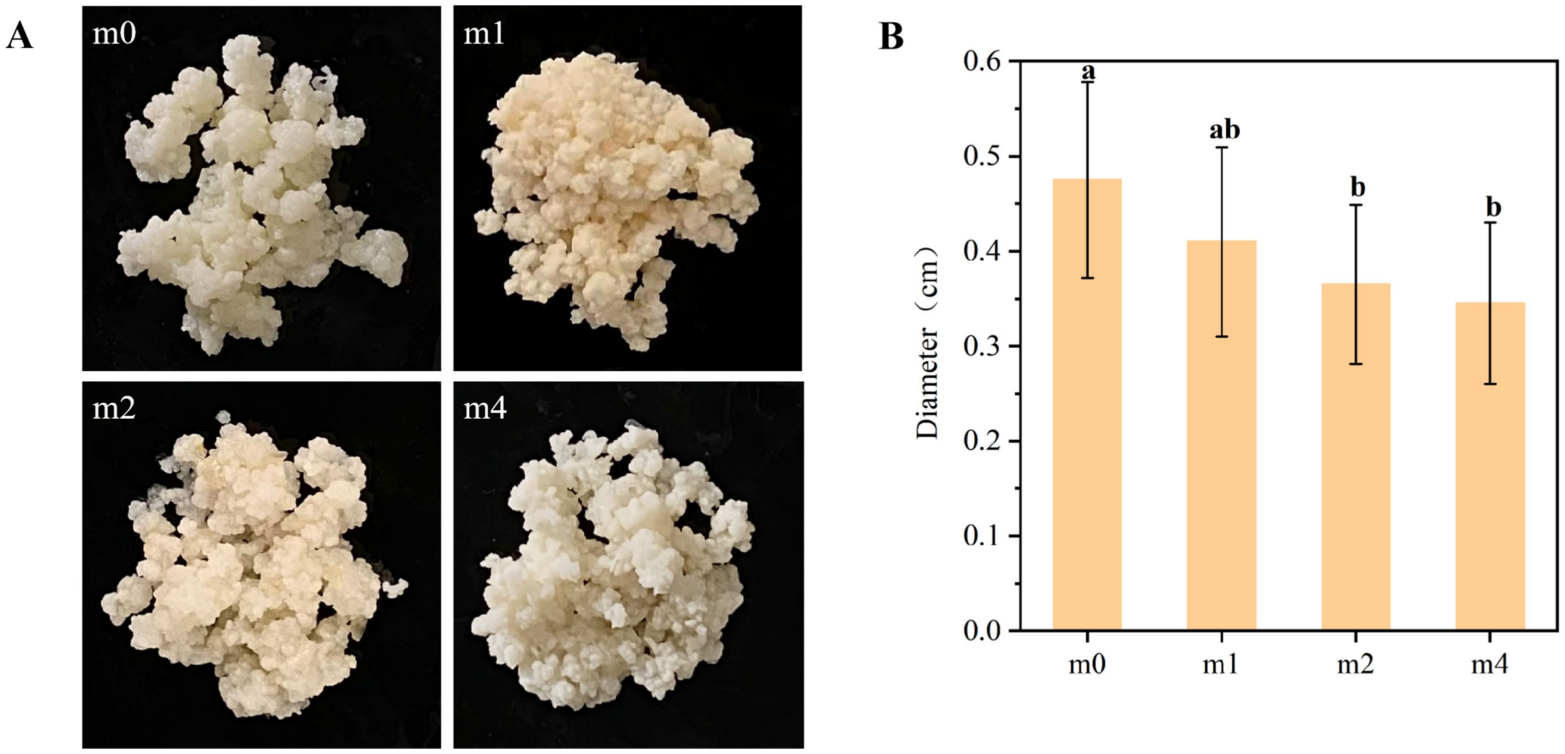
Changes in the macroscopic morphology **(A)** and diameter **(B)** of kefir grains from four subculture time in soymilk. m0, m1, m2, and m4 represent kefir grains subjected to serial culture in soymilk for 0, 1, 2, and 4 months, respectively. The same lowercase letter denotes no significant difference between groups (*p* > 0.05), while different lowercase letters indicate a significant difference (*p* < 0.05).

During subculturing in soymilk, the macroscopic morphology of kefir grains exhibited notable significant alterations in size, color, and viscosity (Figure 5), transitioning from a plump, gelatinous form with non-adhesive surfaces to a less cohesive state. Initially, the kefir grains (m0) were milky white in color, exhibiting a plump, gel-like texture and an average diameter of 0.48 cm (Figure 5B), with no surface stickiness. However, with prolonged subculturing in soymilk, the grains gradually darkened, assuming a yellowish appearance, accompanied by a reduction in diameter, increased surface adhesiveness, and enhanced friability. After 4 months, the average grain diameter significantly decreased to 0.35 cm (*p* < 0.05), and the grains could be readily crushed by hand into a viscous slurry. Consistent with these observations, Mantegazza et al. (2023) reported that handcrafted kefir grains inoculated into a commercial soy beverage and propagated daily through serial subculturing at a 1:50 inoculation ratio no longer visible after several days, yielding a homogeneous, creamy product after 2 weeks. These structural properties of kefir grains are largely attributed to kefiran, an exopolysaccharide (EPS) synthesized primarily by *L. kefiranofaciens* (Ahmed et al., 2013). Kefiran is a unique polymer composed mainly of glucose and galactose in nearly equal proportions (Júnior et al., 2020). This polysaccharide not only imparts the characteristic gel-like texture to kefir grains but also enhances their structural integrity, facilitating the encapsulation of a diverse microbial community (Cheng et al., 2024). Therefore, the progressive disintegration of kefir grain structure observed during serial subculturing in soymilk may be directly related to the decline in *L. kefiranofaciens* abundance, which in turn leads to reduced kefiran production and compromised structural stability.

### 3.3 Microscopic morphology

**Figure 6:**
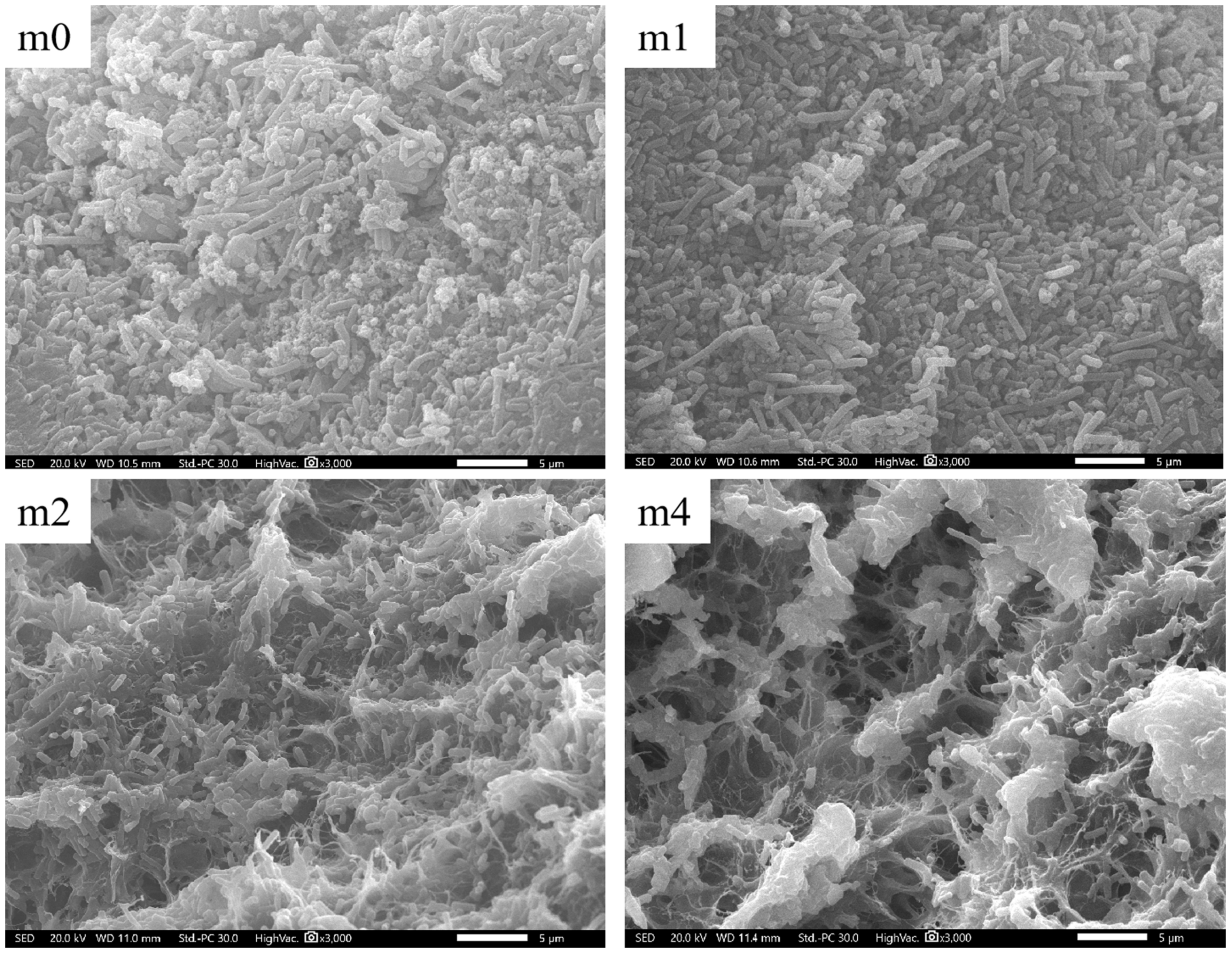
Changes in microscopic morphology of kefir grains from four subculture time in soymilk (magnification: 3000×). m0, m1, m2, and m4 represent kefir grains subjected to serial culture in soymilk for 0, 1, 2, and 4 months, respectively. The white scale bar (−) represents 5 μm.

SEM observations revealed significant variations in microbial distribution and extracellular matrix architecture on the surface of kefir grains across the four successive subculturing periods in soymilk (Figure 6). At the initial stage (m0), the grain surface exhibited a densely packed network predominantly composed of long rod-shaped bacterial cells, interspersed with embedded yeast cells. Consistent with our findings, Wang et al. (2018) previously reported that *L. kefiranofaciens* predominantly exhibits a long-rod morphology on the surface of kefir grains. Given that *L. kefiranofaciens* was the dominant species in kefir grains at the m0 stage (Figure 2D), the abundance of long-rod-shaped bacteria observed on the surface of m0 grains could be attributed to *L. kefiranofaciens*. The microbial consortium was enmeshed within an extracellular matrix rich in EPS and proteins, forming a cohesive and continuous biofilm-like structure. With subculturing progression, the matrix integrity gradually deteriorated. By the fourth month (m4), the microbial landscape underwent marked structural reorganization. With the succession of the microbial community, long rod-shaped bacteria were largely replaced by short rod-shaped morphotypes resulting in a sparsely distributed microbial network. The originally homogeneous matrix transitioned into a reticulated, porous architecture characterized by irregular void formation. Meanwhile, surface-adhered extracellular polymeric substances (EPS) exhibited reduced coverage and a fragmented spatial organization compared to the initial state (m0). This structural transformation aligns with microbial succession dynamics, where the shift in dominance from *L. kefiranofaciens* (a long-rod EPS producer) to *L. paracasei* [a short-rod (Sun et al., 2022)] likely disrupted the EPS-dependent biofilm framework. Consequently, the destabilization of this matrix led to its fragmentation and the emergence of porous structures.

### 3.4 Changes in the fermentation characteristics of kefir grains during subculturing

#### 3.4.1 pH

**Figure 7:**
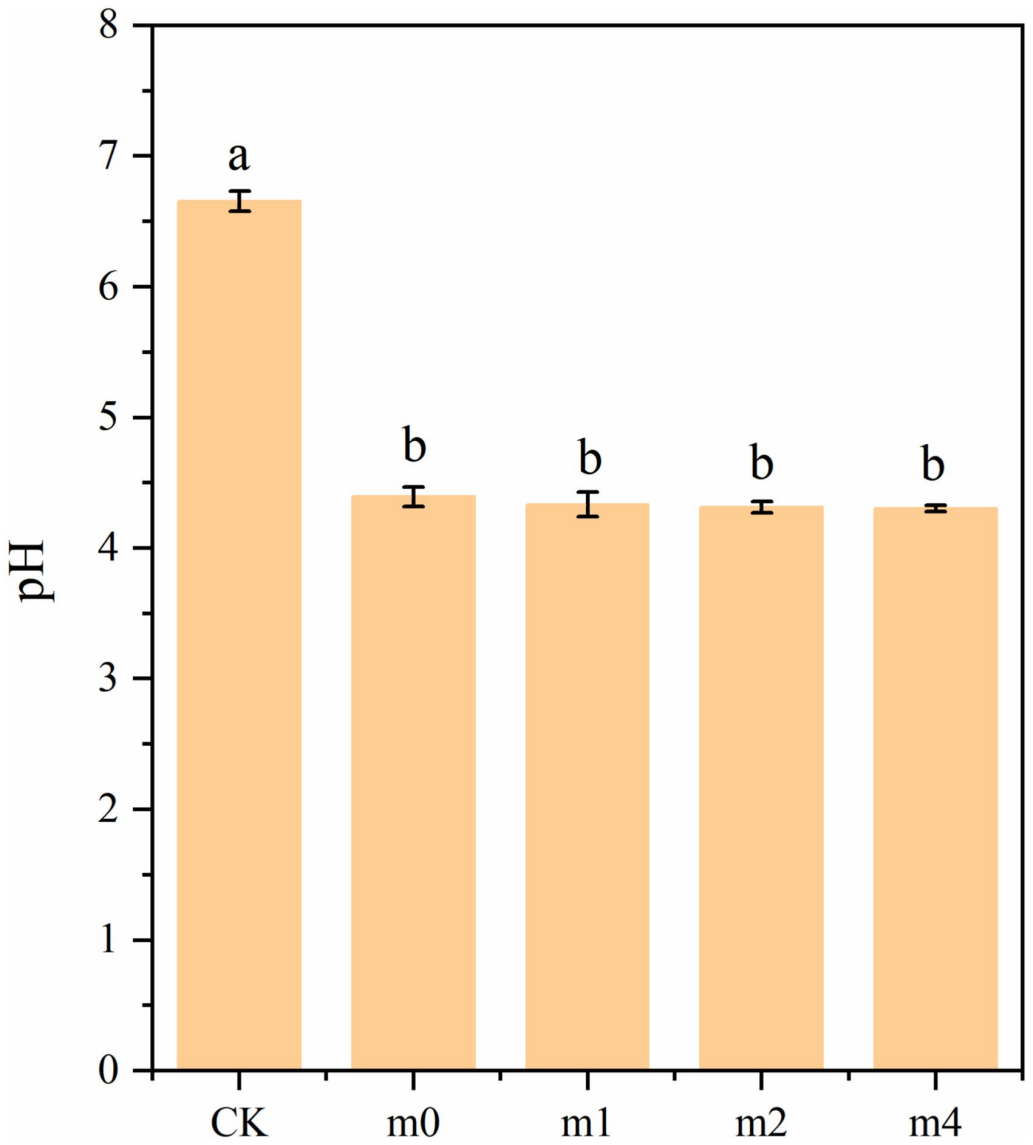
pH of soymilk fermented with the four different kefir grains. m0, m1, m2, and m4 represent kefir grains subjected to serial culture in soymilk for 0, 1, 2, and 4 months, respectively. The same lowercase letter denotes no significant difference between groups (*p* > 0.05), while different lowercase letters indicate a significant difference (*p* < 0.05).

Changes in pH during soymilk fermentation with the four different kefir grain communities are presented in Figure 7. The pH of unfermented control soymilk (CK) was 6.65. Following 24 h of fermentation, all fermented samples exhibited significant acidification (*p* < 0.05), with final pH values of 4.39 (m0), 4.33 (m1), 4.31 (m2), and 4.30 (m3). Although pH values exhibited a gradual decreasing trend with increasing subculture generations, no statistically significant differences (*p* > 0.05) were detected among the fermented samples. pH is a crucial factor influencing the stability, aroma, flavor, and texture of fermented soymilk products (dos Santos et al., 2019). An optimal pH range of 4.2 ~ 4.5 is generally recommended for fermented soymilk to promote desirable gel formation, enhance resistance to spoilage, and impart a mildly acidic, refreshing taste characteristic of this product (Tavşanlı et al., 2024). The pH values of all four samples fell within this optimal range.

#### 3.4.2 Sensory evaluation

**Figure 8:**
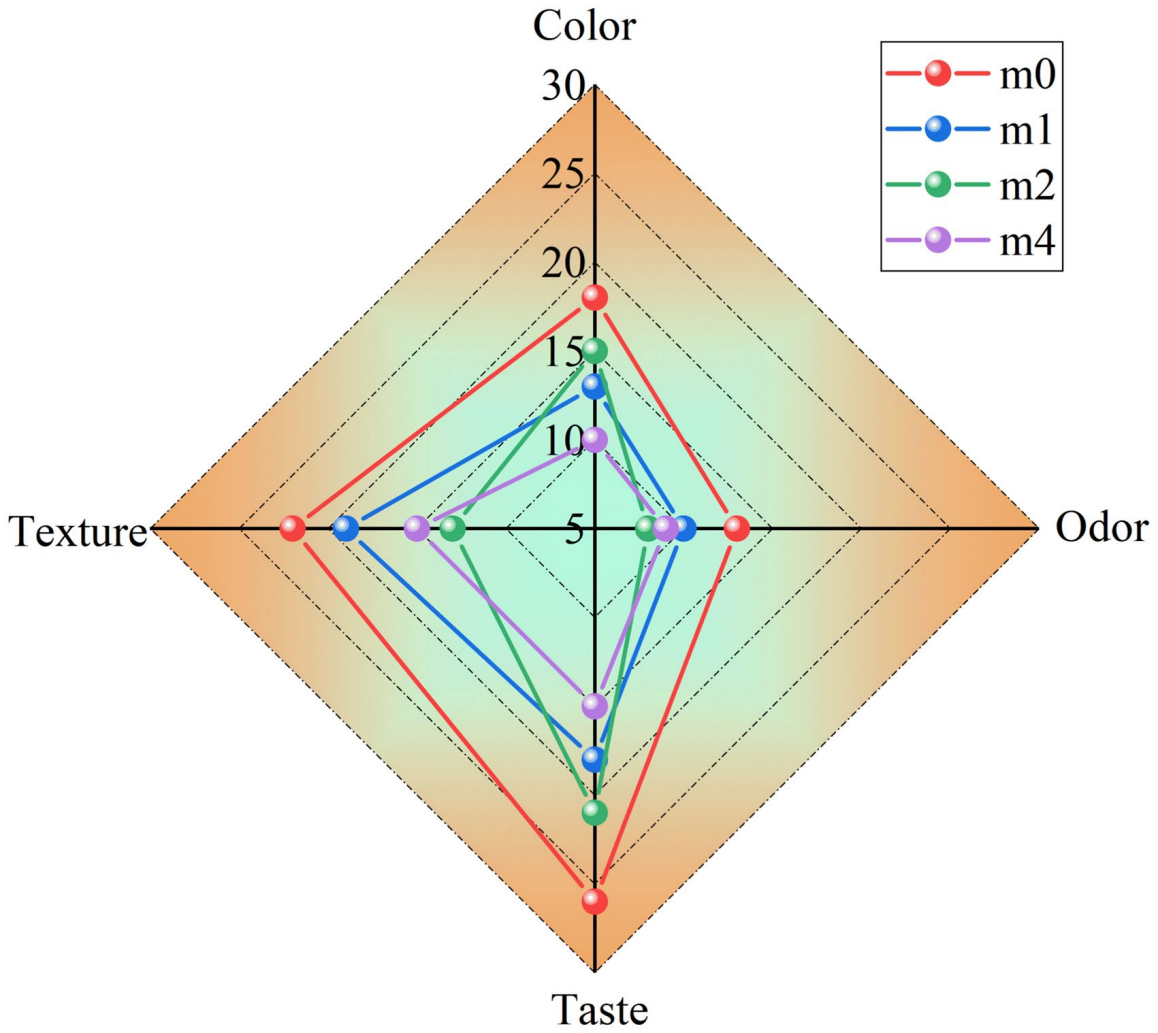
Sensory evaluation scores of soymilk fermented with the four different kefir grains. m0, m1, m2, and m4 represent kefir grains subjected to serial culture in soymilk for 0, 1, 2, and 4 months, respectively.

Significant alterations in the sensory characteristics of fermented soymilk were observed during the four-month successive subculturing of kefir grains in soymilk (Figure 8). The initial fermentation (m0) yielded a product with a uniform milky-white appearance and a characteristic fermented aroma. This product exhibited desirable textural properties, characterized by a smooth mouthfeel, creamy consistency, and a complete absence of whey separation. However, progressive deterioration of sensory quality was detected with increasing subculture duration. In later generations, the fermented soymilk exhibited compromised color homogeneity, a diminished aromatic profile, increased whey exudation, and textural defects such as graininess and reduced viscosity. *L. kefiranofaciens*, which are dominant in traditional kefir grains, play a critical role in maintaining microbial homeostasis and suppressing the growth of undesirable bacteria (Blasche et al., 2021). However, during successive subculturing of kefir grains in soymilk, the relative abundance of *L. kefiranofaciens* declined sharply, while *L. paracasei* gradually became dominant. This shift in the microbial community structure weakened the regulatory capacity of the original kefir microbiota. Consequently, there was a notable increase in the relative abundance of environmental and opportunistic bacteria, including *Stenotrophomonas maltophilia*, *Pseudomonas putida*, *Pseudomonas fulva*, *Enterococcus casseliflavus* and *Enterococcus durans* (Figure 2). The resulting increase in microbial complexity disrupted the cooperative interactions among key fermentative strains, ultimately contributing to the deterioration of the sensory quality of the fermented soymilk.

### 3.5 Amino nitrogen content

**Figure 9:**
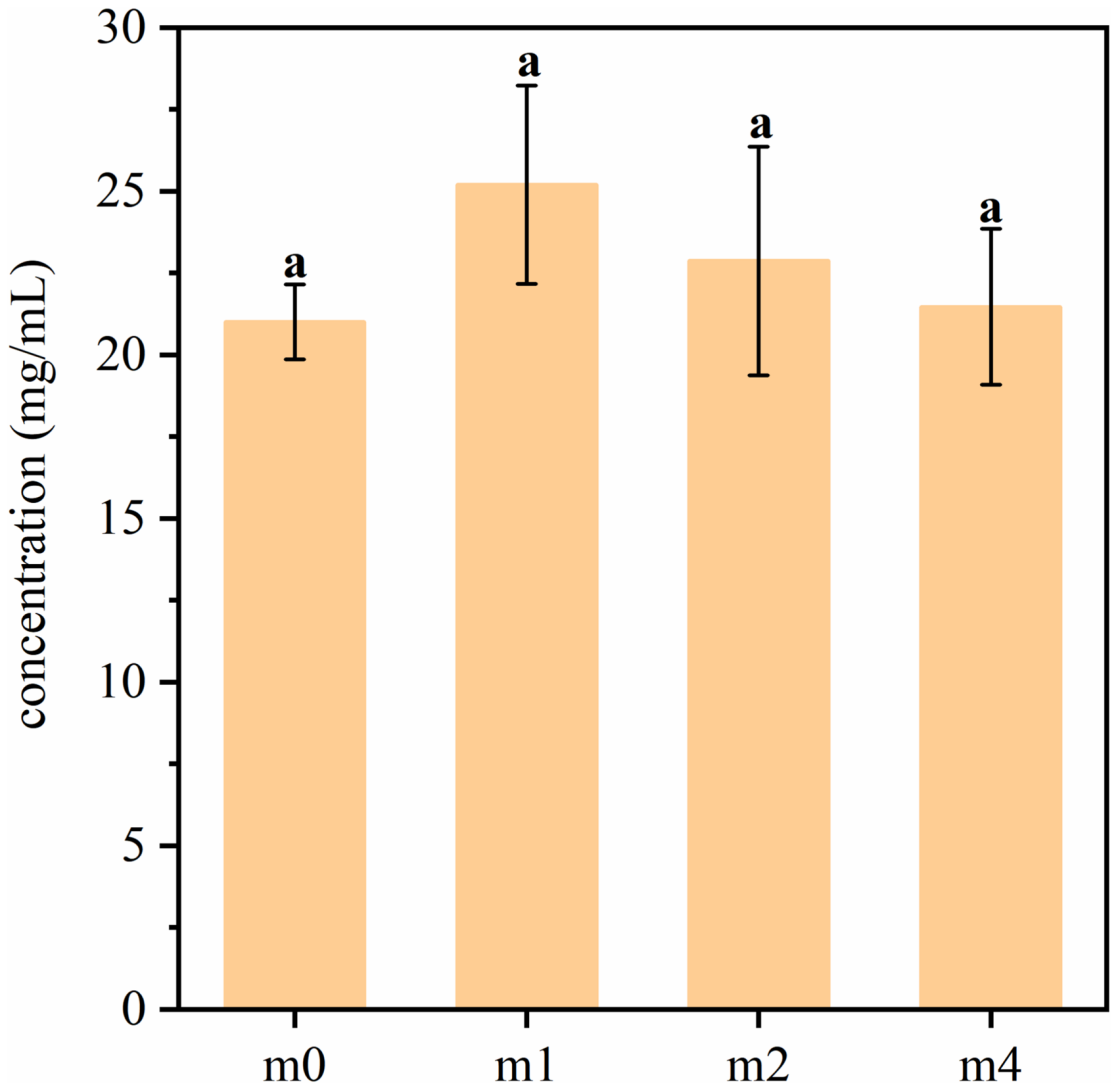
Amino-peptide nitrogen content in soymilk fermented with the four different kefir grains. m0, m1, m2, and m4 represent kefir grains subjected to serial culture in soymilk for 0, 1, 2, and 4 months, respectively. The same lowercase letter denotes no significant difference between groups (*p* > 0.05), while different lowercase letters indicate a significant difference (*p* < 0.05).

The amino-peptide nitrogen content in soymilk fermented with the four kefir grain communities is presented in Figure 9. Statistical analysis revealed no significant differences (*p* > 0.05) in amino-peptide nitrogen levels among the four fermented soymilk samples. This consistent pattern across different kefir cultures suggests that the proteolytic activity leading to amino-peptide generation remains relatively stable, irrespective of the specific kefir grain community, under the conditions tested. *L. paracasei* is known for its relatively strong proteolytic activity (Du et al., 2024), during successive subculturing of kefir grains in soymilk, the abundance of traditional kefir dominant strains such as *L. kefiranofaciens* and *L. kefiri* decreased, which may have led to a loss of some key proteolytic functions. However, the enrichment of *L. paracasei* appears to have compensated for this shift, maintaining the overall protein degradation capacity of the fermentation system.

### 3.6 Antioxidant activity and ACE inhibitory activity

**Figure 10:**
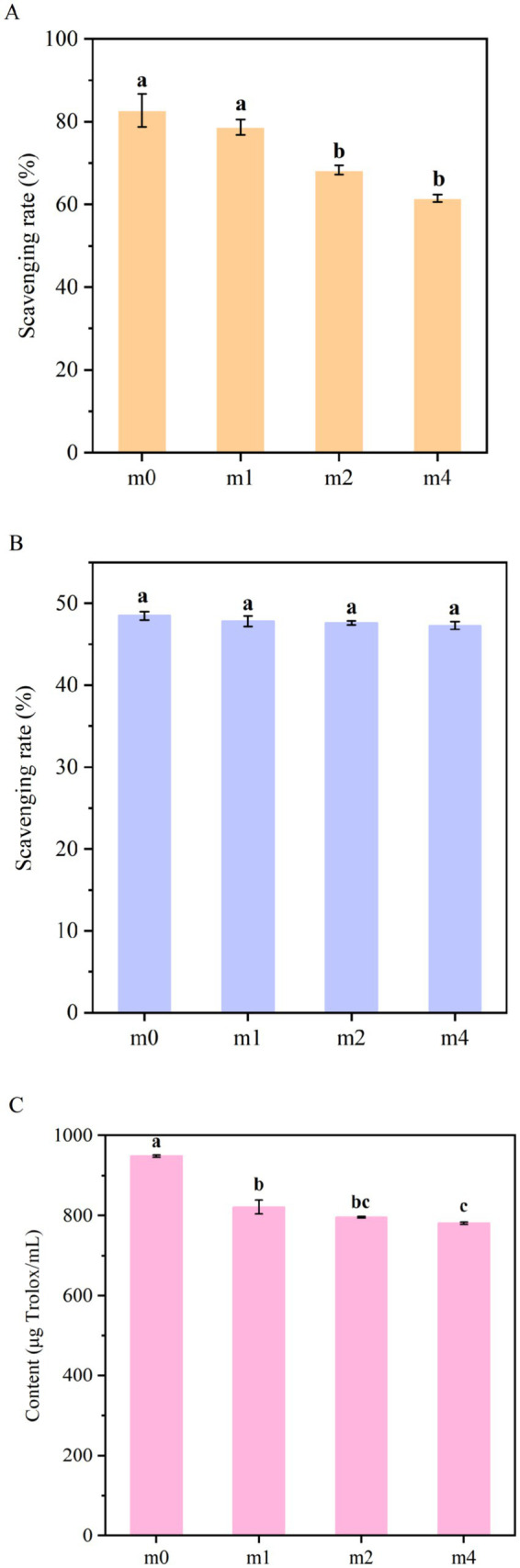
Antioxidant activity of soymilk fermented with the four different kefir grains. **(A)** ABTS, **(B)** DPPH, **(C)** FRAP. m0, m1, m2, and m4 represent kefir grains subjected to serial culture in soymilk for 0, 1, 2, and 4 months, respectively. The same lowercase letter denotes no significant difference between groups (*p* > 0.05), while different lowercase letters indicate a significant difference (*p* < 0.05).

The antioxidant capacities of the four fermented soymilk samples were systematically evaluated using the ABTS radical scavenging assay, the DPPH radical scavenging assay, and the ferric reducing antioxidant power (FRAP) assay (Figure 10). Statistical analysis revealed significant time-dependent decreases in both ABTS radical scavenging capacity and ferric reducing power with prolonged subculture duration. Following 4 months of successive subculturing, the ABTS radical scavenging activity decreased significantly from 82.4 to 61.5% (*p* < 0.05), while the FRAP value decreased significantly from 948.63 μg Trolox/mL to 781.3 μg Trolox/mL (*p* < 0.05). In contrast, the DPPH radical scavenging activity showed only a slight decline from 48.47 to 47.28%, with no statistically significant difference observed during the experimental period (*p* > 0.05).

**Figure 11:**
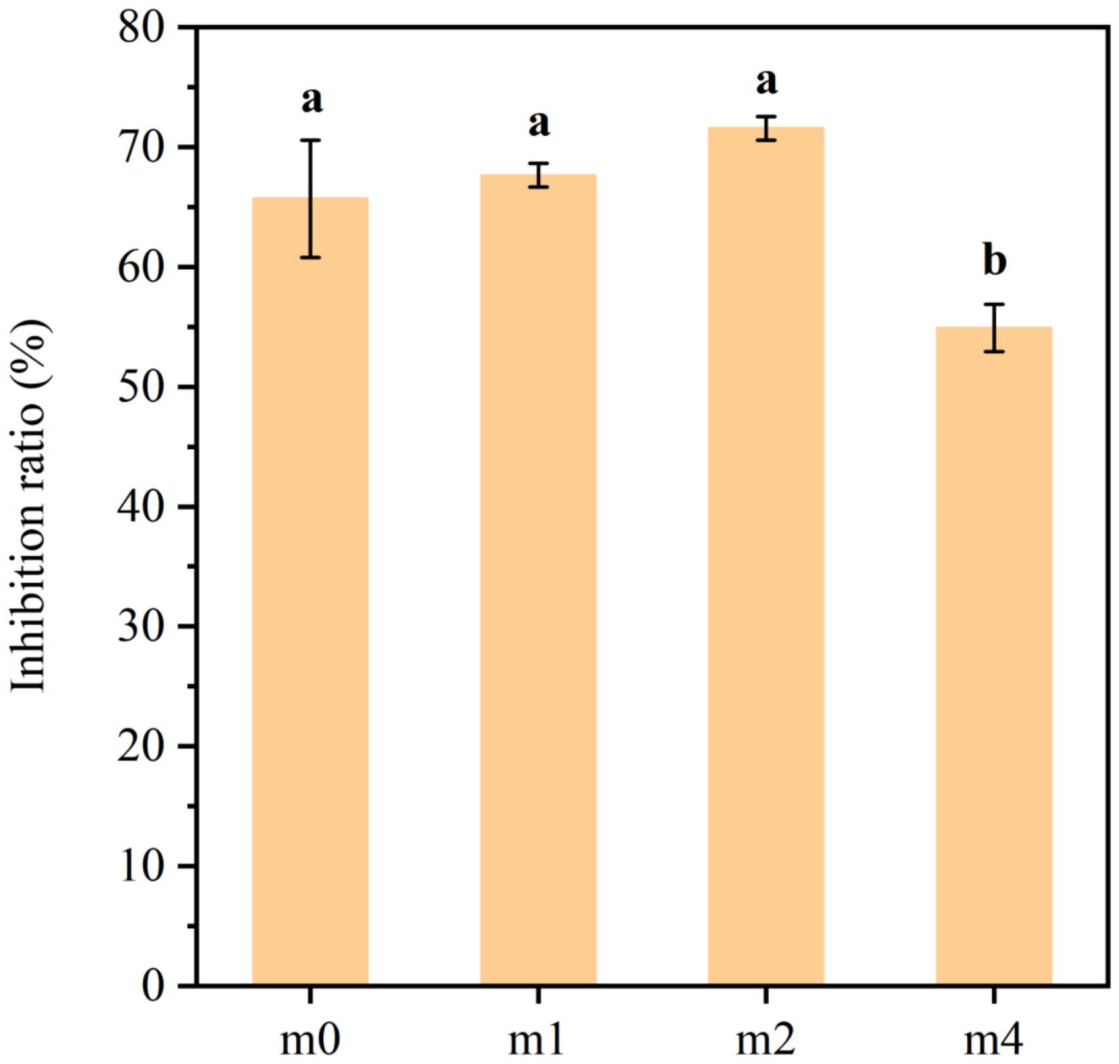
ACE inhibitory activity of soymilk fermented with the four different kefir grains. m0, m1, m2, and m4 represent kefir grains subjected to serial culture in soymilk for 0, 1, 2, and 4 months, respectively. The same lowercase letter denotes no significant difference between groups (*p* > 0.05), while different lowercase letters indicate a significant difference (*p* < 0.05).

The ACE inhibitory activity of soymilk fermented with the four distinct kefir grain communities is illustrated in Figure 11. The results demonstrated that kefir grain-fermented soymilk exhibited potent ACE inhibitory activity at the start of the experiment (m0: 65.7%), and remained relatively stable throughout most of the successive subculturing. However, a statistically significant decrease in ACE inhibitory activity was observed after 4 months of subculturing (m4: 54.9%, *p* < 0.05), indicating a gradual loss of functional potential with prolonged subculture.

In the early passages, *L. kefiranofaciens and L. kefiri* were dominant and likely contributed to the synthesis of bioactive compounds including antioxidant peptides (Silva et al., 2024) and exopolysaccharides (Jeong et al., 2017). However, due to the limited adaptability of these core kefir microbes to the soymilk environment, their abundance decreased over time. Although *L. paracasei* became more dominant and partially compensated for protein hydrolysis, it may not have produced the same potency of bioactive peptides as the original community. Moreover, the increased presence of non-beneficial or opportunistic species, such as *S. maltophilia*, *P. putida*, *P. fulva*, *E. casseliflavus* and *E. durans* may have disrupted the cooperative metabolic networks essential for bioactivity generation. As a result, despite stable levels of amino nitrogen, the antioxidant activity and ACE inhibitory activity of the fermented soymilk was significantly compromised.

## 4 Conclusion

Successive subculturing of kefir grains in soymilk for 4 months resulted in notable alterations in the microbial community composition. The relative abundance of *L. kefiranofaciens* decreased significantly from 95.00 to 15.70%, while *L. paracasei* increased from 0.32 to 76.94%. The predominance of *L. paracasei* is attributed to its possession of key enzymes involved in the metabolism of raffinose, stachyose, and sucrose, facilitating efficient utilization of these oligosaccharides. The decline in *L. kefiranofaciens*, a major producer of polysaccharides essential for maintaining the structural integrity of kefir grains, led to reduced polysaccharide content, resulting in smaller grain diameter, increased surface viscosity, and partial disintegration of the gel-like structure. Concurrently, although the pH and free amino acid content of the fermented soymilk remained relatively stable, sensory evaluation scores decreased, along with a reduction in antioxidant activity and ACE inhibitory activity.

These results indicate that soymilk is a viable substrate for kefir grains fermentation, supporting both microbial growth and bioactive compound production. However, prolonged subculturing negatively impacts the stability and functionality of the grains. Therefore, we recommend limiting continuous propagation of kefir grains in soymilk to a maximum of 1 month. Regular renewal of kefir grains is essential to maintain grain integrity and ensure consistent product quality during long-term production.

## Data Availability

The datasets presented in this study can be found in online repositories. The names of the repository/repositories and accession number(s) can be found in the article/supplementary material.

## Footnotes

^1^ https://www.bioincloud.tech/

## References

[ref1] Ahmed Z. Wang Y. Anjum N. Ahmad A. Khan S. T. (2013). Characterization of exopolysaccharide produced by *Lactobacillus kefiranofaciens* ZW3 isolated from Tibet kefir-part II. Food Hydrocoll. 30, 343–350. doi: 10.1016/j.foodhyd.2012.06.009

[ref2] Alraddadi F. A. Ross T. Powell S. M. (2023). Evaluation of the microbial communities in kefir grains and kefir over time. Int. Dairy J. 136:105490. doi: 10.1016/j.idairyj.2022.105490

[ref3] Aspri M. Leni G. Galaverna G. Papademas P. (2018). Bioactive properties of fermented donkey milk, before and after in vitro simulated gastrointestinal digestion. Food Chem. 268, 476–484. doi: 10.1016/j.foodchem.2018.06.119, PMID: 30064786

[ref4] Azizi N. F. Kumar M. R. Yeap S. K. Abdullah J. O. Khalid M. Omar A. R.. (2021). Kefir and its biological activities. Food Secur. 10:1210. doi: 10.3390/foods10061210, PMID: 34071977 PMC8226494

[ref5] Barãoa C. E. Klososkia S. J. Pinheiroa K. H. Marcolinoa V. A. Juniorb O. V. da Cruzc A. G.. (2019). Growth kinetics of kefir biomass: influence of the incubation temperature in milk. Chem. Eng. 75, 499–504. doi: 10.3303/CET1975084

[ref6] Baú T. Garcia S. Ida E. (2015). Changes in soymilk during fermentation with kefir culture: oligosaccharides hydrolysis and isoflavone aglycone production. Int. J. Food Sci. Nutr. 66, 845–850. doi: 10.3109/09637486.2015.1095861, PMID: 26460145

[ref7] Blasche S. Kim Y. Mars R. A. Machado D. Maansson M. Kafkia E.. (2021). Metabolic cooperation and spatiotemporal niche partitioning in a kefir microbial community. Nat. Microbiol. 6, 196–208. doi: 10.1038/s41564-020-00816-5, PMID: 33398099 PMC7610452

[ref8] Chen Z. Cao S. Yin L. Shao M. Ye T. Xu Z.. (2025). Safety and probiotic evaluation of *Lactiplantibacillus plantarum* CHEN1 and its metabolic analysis of soybean oligosaccharides. Sci. Technol. Food Ind. 46, 202–211. doi: 10.13386/j.issn1002-0306.2024110347

[ref9] Chen Z. Shi J. Yang X. Nan B. Liu Y. Wang Z. (2015). Chemical and physical characteristics and antioxidant activities of the exopolysaccharide produced by Tibetan kefir grains during milk fermentation. Int. Dairy J. 43, 15–21. doi: 10.1016/j.idairyj.2014.10.004

[ref10] dos Santos D. C. de Oliveira Filho J. G. Santana A. C. A. de Freitas B. S. M. Silva F. G. Takeuchi K. P.. (2019). Optimization of soymilk fermentation with kefir and the addition of inulin: physicochemical, sensory and technological characteristics. LWT 104, 30–37. doi: 10.1016/j.lwt.2019.01.030

[ref11] Du X. Yin S. Wang T. Chu C. Devahastin S. Yi J.. (2024). Identification of proteolytic bacteria from Yunnan fermented foods and their use to reduce the allergenicity of β-lactoglobulin. J. Dairy Sci. 107, 8990–9004. doi: 10.3168/jds.2024-25055, PMID: 39004134

[ref12] Egea M. B. Santos D. Neves J. F. Lamas I. B. Oliveira-Filho J. Takeuchi K. (2023). Physicochemical characteristics and rheological properties of soymilk fermented with kefir. Biointerface Res. Appl. Chem. 13, 1–10. doi: 10.33263/briac132.127

[ref13] Cheng T. Zhang T. Zhang P. He X. Sadiq F. A. Li J.. (2024). The complex world of kefir: structural insights and symbiotic relationships. Compr. Rev. Food Sci. Food Saf. 23:e13364. doi: 10.1111/1541-4337.13364, PMID: 38847746

[ref14] Gao J. Gu F. Abdella N. H. Ruan H. He G. (2012). Investigation on culturable microflora in Tibetan kefir grains from different areas of China. J. Food Sci. 77, M425–M433. doi: 10.1111/j.1750-3841.2012.02805.x, PMID: 22860591

[ref15] Garofalo C. Ferrocino I. Reale A. Sabbatini R. Milanović V. Alkić-Subašić M.. (2020). Study of kefir drinks produced by backslopping method using kefir grains from Bosnia and Herzegovina: microbial dynamics and volatilome profile. Food Res. Int. 137:109369. doi: 10.1016/j.foodres.2020.109369, PMID: 33233071

[ref16] Gökırmaklı Ç. Güzel-Seydim Z. B. (2022). Water kefir grains vs. milk kefir grains: physical, microbial and chemical comparison. J. Appl. Microbiol. 132, 4349–4358. doi: 10.1111/jam.15532, PMID: 35301787

[ref17] Gul O. Mortas M. Atalar I. Dervisoglu M. Kahyaoglu T. (2015). Manufacture and characterization of kefir made from cow and buffalo milk, using kefir grain and starter culture. J. Dairy Sci. 98, 1517–1525. doi: 10.3168/jds.2014-8755, PMID: 25582588

[ref18] Gut A. M. Vasiljevic T. Yeager T. Donkor O. N. (2019). Characterization of yeasts isolated from traditional kefir grains for potential probiotic properties. J. Funct. Foods 58, 56–66. doi: 10.1016/j.jff.2019.04.046

[ref19] Jeong D. Kim D. H. Kang I. B. Kim H. Song K. Y. Kim H. S.. (2017). Characterization and antibacterial activity of a novel exopolysaccharide produced by *Lactobacillus kefiranofaciens* DN1 isolated from kefir. Food Control 78, 436–442. doi: 10.1016/j.foodcont.2017.02.033

[ref20] Júnior L. M. Vieira R. P. Anjos C. A. R. (2020). Kefiran-based films: fundamental concepts, formulation strategies and properties. Carbohydr. Polym. 246:116609. doi: 10.1016/j.carbpol.2020.116609, PMID: 32747252

[ref21] Ketnawa S. Ogawa Y. (2021). In vitro protein digestibility and biochemical characteristics of soaked, boiled and fermented soybeans. Sci. Rep. 11:14257. doi: 10.1038/s41598-021-93451-x, PMID: 34244542 PMC8270925

[ref22] Li M. Xia S. Zhang Y. Li X. (2018). Optimization of ACE inhibitory peptides from black soybean by microwave-assisted enzymatic method and study on its stability. LWT 98, 358–365. doi: 10.1016/j.lwt.2018.08.045

[ref23] Liu J. R. Lin C. W. (2000). Production of kefir from soymilk with or without added glucose, lactose, or sucrose. J. Food Sci. 65, 716–719. doi: 10.1111/j.1365-2621.2000.tb16078.x

[ref24] Long N. Liu J. Liu J. Li Y. Hou Y. Liao X.. (2022). Single-molecule real-time sequencing to explore the mycobiome diversity in malt. Microbiol. Spectr. 10:e00511-00522. doi: 10.1128/spectrum.00511-22, PMID: 36154437 PMC9603040

[ref25] Luo J. Liu S. Lu H. Chen Q. Shi Y. (2023). Microbial community variations and bioconversion improvements during soybean-based fermentation by kefir grains. Food Secur. 12:1588. doi: 10.3390/foods12081588, PMID: 37107383 PMC10137332

[ref26] Mantegazza G. Dalla Via A. Licata A. Duncan R. Gardana C. Gargari G.. (2023). Use of kefir-derived lactic acid bacteria for the preparation of a fermented soy drink with increased estrogenic activity. Food Res. Int. 164:112322. doi: 10.1016/j.foodres.2022.112322, PMID: 36737914

[ref27] Porebski S. Bailey L. G. Baum B. R. (1997). Modification of a CTAB DNA extraction protocol for plants containing high polysaccharide and polyphenol components. Plant Mol. Biol. Report. 15, 8–15. doi: 10.1007/bf02772108

[ref28] Purutoğlu K. İspirli H. Yüzer M. O. Serencam H. Dertli E. (2020). Diversity and functional characteristics of lactic acid bacteria from traditional kefir grains. Int. J. Dairy Technol. 73, 57–66. doi: 10.1111/1471-0307.12633

[ref29] Re R. Pellegrini N. Proteggente A. Pannala A. Yang M. Rice-Evans C. (1999). Antioxidant activity applying an improved ABTS radical cation decolorization assay. Free Radic. Biol. Med. 26, 1231–1237. doi: 10.1016/s0891-5849(98)00315-3, PMID: 10381194

[ref30] Silva M. H. Batista L. L. Malta S. M. Santos A. C. Mendes-Silva A. P. Bonetti A. M.. (2024). Unveiling the Brazilian kefir microbiome: discovery of a novel *Lactobacillus kefiranofaciens* (LkefirU) genome and in silico prospection of bioactive peptides with potential anti-Alzheimer properties. BMC Genomics 25:884. doi: 10.1186/s12864-024-10695-3, PMID: 39304820 PMC11414172

[ref31] Sun Y. Wang H. Zhou L. Chang M. Yue T. Yuan Y.. (2022). Distribution characteristics of organic selenium in se-enriched *Lactobacillus* (*Lactobacillus paracasei*). LWT 165:113699. doi: 10.1016/j.lwt.2022.113699

[ref32] Tavşanlı N. Yıldız S. Çalışkan M. Aydin S. (2024). Evaluating the potential effect of microalgae on soymilk vegan kefir in terms of physical, chemical, microbiological properties. Algal Res. 82:103630. doi: 10.1016/j.algal.2024.103630

[ref33] Wang H. Wang C. Guo M. (2020). Autogenic successions of bacteria and fungi in kefir grains from different origins when sub-cultured in goat milk. Food Res. Int. 138:109784. doi: 10.1016/j.foodres.2020.109784, PMID: 33288170

[ref34] Wang X. Xiao J. Jia Y. Pan Y. Wang Y. (2018). *Lactobacillus kefiranofaciens*, the sole dominant and stable bacterial species, exhibits distinct morphotypes upon colonization in Tibetan kefir grains. Heliyon 4:e00649. doi: 10.1016/j.heliyon.2018.e00649, PMID: 30009271 PMC6042379

[ref35] Zanirati D. F. Abatemarco Jr M. de Cicco Sandes S. H. Nicoli J. R. Nunes Á. C. Neumann E. (2015). Selection of lactic acid bacteria from Brazilian kefir grains for potential use as starter or probiotic cultures. Anaerobe 32, 70–76. doi: 10.1016/j.anaerobe.2014.12.007 25542841

[ref36] Zeng X. Wang Y. Jia H. Wang Z. Gao Z. Luo Y.. (2022). Metagenomic analysis of microflora structure and functional capacity in probiotic Tibetan kefir grains. Food Res. Int. 151:110849. doi: 10.1016/j.foodres.2021.110849, PMID: 34980387

